# Oral Mucositis in Cancer and Potential Use of Omega-3 Free Fatty Acids in Its Management: A Review

**DOI:** 10.3390/biomedicines9111531

**Published:** 2021-10-25

**Authors:** Roberta Cardim Lessa, Fabio de Abreu Alves, Erika Fortunati, Jun Lu

**Affiliations:** 1 School of Science, Faculty of Health and Environmental Sciences, Auckland University of Technology, Auckland 1142, New Zealand; roberta.lessa@aut.ac.nz (R.C.L.); pjz3731@autuni.ac.nz (E.F.); 2 Department of Stomatology, A.C. Camargo Cancer Center, FO-USP, São Paulo 01525-001, Brazil; falves@accamargo.org.br; 3 School of Public Health and Interdisciplinary Studies, Faculty of Health and Environmental Sciences, Auckland University of Technology, Auckland 1142, New Zealand; 4 College of Food Engineering and Nutrition Sciences, Shaanxi Normal University, Xi’an 710119, China; 5 College of Food Science and Technology, Nanchang University, Nanchang 330031, China; 6 Maurice Wilkins Centre for Molecular Discovery, Auckland 1010, New Zealand

**Keywords:** oral mucositis, inflammation, polyunsaturated fatty acids, omega-3 fatty acids, omega-6 fatty acids, cancer, prostaglandins, leukotrienes, interleukins, cytokines

## Abstract

Oral mucositis (OM) is a painful condition caused by chemotherapeutic or radiotherapeutic cancer treatments, occurring in patients with different tumour characteristics and locations. OM greatly impacts a patient’s quality of life and cancer recovery. Current OM management strategies are not providing sufficient prevention and treatment; new approaches to injury management are needed. Studies on the benefit of omega-3 free fatty acids (FFA) in human health have increased significantly in recent years. FFA properties have been studied extensively, including their potential therapeutic use in inflammatory conditions. However, omega-3 FFA’s use as a supplementary treatment for OM has not been clinically tested. Preliminary evidence suggests that utilising FFA to manage OM could be a useful strategy for lesion management, assisting with healthy oral mucosa recovery. This review will describe the incidence, risk factors, biology of OM and the current treatment strategies, leading to a discussion of the utility of omega-3 FFA as a novel therapeutic agent for OM.

## 1. Introduction

Oral mucositis (OM) is a severe form of acute inflammation and ulceration in the oral mucosa that can be induced by oncological therapy. OM induces erythema and swelling in the oral mucosa, followed by generalised ulceration and bleeding that can spread further from the oral cavity to the digestive tract and that is capable of causing debilitating effects for patients with OM. Effects include pain, inflammation, compromised oral hygiene, an increased risk for local and systemic infections as well as impaired nutrition [1,2,3,4]. The OM doubles the risk of systemic infections and quadruples the risk of death in cancer patients. Supportive care approaches to managing symptoms are commonly used in the OM condition. However, due the complicated pathobiology, those interventions are often not efficient and effective for all patients. Therefore, OM relief still represents an unmet need.

The aim of this review is to update knowledge about the concept, incidence and pathogenesis of OM, to examine the well-established therapeutic strategies in the prevention or treatment of OM and to discuss how omega-3 free fatty acids supplements could be used to treat OM patients.

## 2. Incidence and Risk Assessment

The incidence of OM in patients with various types of cancer ranges from approximately 30% to 40% to almost 100% [5]. However, the most severe and debilitating type of OM observed in cancer patients is caused by head and neck radiation therapy, where it manifests in almost all patients [6]. OM develops in approximately 60% to 85% of patients undergoing hematopoietic stem cell transplantation (HSCT) and 20% to 40% of patients receiving conventional chemotherapies [7]. The use of concomitant chemotherapy and targeted agents increases OM risk [5]. The true prevalence of OM as an adverse effect in oncological treatment has potentially been underreported. OM incidence and severity vary depending on the treatment modality. Often, its severity is clinically observed in patients receiving chemotherapy, radiotherapy, or a combination of the two. Radio-induced OM occurs in head and neck cancer patients who receive cumulative radiation doses ranging from <32 gray (Gy) to greater than 65 Gy. However, dose fractionation protocols and differences in RT techniques result in different incidence rates [8]. Add to these variables the locations and intensities of OM that are associated with the vast range of chemotherapeutic drugs. The highest incidence of OM occurs in patients that receive antimetabolites, platin-derived DNA abductors, taxanes, anthracyclines, irinotecan and alkylating agents [9]. More research is required to understand the true prevalence of OM pathology in oncological therapies.

OM adversely affects several clinical outcomes for patients. Cancer patients who develop this comorbidity typically experience a decreased tolerance for therapy, are at a higher risk of readmission and tend to have longer hospitalisation periods than patients without OM [10]. An intensive care unit study revealed that the most commonly diagnosed alterations in the oral cavity were an imbalance in the oral microbiota, oral candidiasis, salivary flow changes and mucositis [11]. Furthermore, OM negatively affects the nutritional status of cancer patients since dysphagia (difficulty in feeding) with solid and liquid food, dysarthria (poor coordination of the speech muscles) and odynophagia (pain or burning sensation when swallowing) affect food intake and other nutritional supplementation [12]. Additionally, symptoms related to developmental pathology, from lower-grade oral burning to severe pain and spontaneous bleeding that disrupts routine feeding, may lead to cachexia (the loss of skeletal muscle and fat tissues), requiring parenteral nutrition via a nasogastric tube [2].

Comorbidities alongside OM can lead to severe systemic disorders, immunosuppression and even sepsis [1,3,7]. Therefore, many studies have been seeking strategies to aid faster recoveries of inflammatory pathological conditions by reducing the rate of oral cavity contamination by harmful microorganisms [11]. OM is a prevalent, adverse effect of cancer treatment that has likely been underreported despite its negative ramifications in patients’ lives [12]. As a consequence, considerable research is being carried out to determine agents and strategies which promote the prevention of OM and the recovery from its disruptions to patients’ quality of life.

## 3. Biomolecular Mechanisms of OM

**Figure 1:**
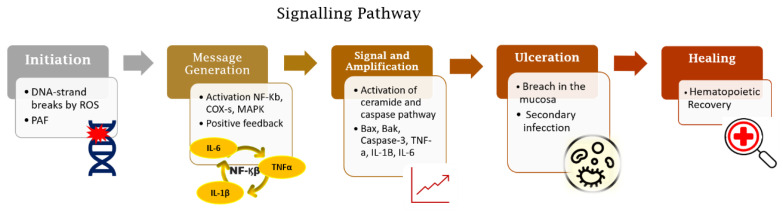
Five-phase pathobiological model of oral mucositis. Based on this model, the process trigger is DNA damage induced by radio-chemotherapy followed by activation of inflammatory pathways together with apoptosis. These processes lead to the loss of integrity of the mucosal barrier and subsequent wound formation. The end of the signalling pathway occurs spontaneously after cessation of tissue damage.

Radiation-induced and chemotherapy-induced OM have similar developmental mechanisms [1]. The cascade of biological events responsible for the genesis of OM begins with the induction of DNA damage caused by radiation or chemotherapeutic cancer therapy [6,13] (Figure 1).

**Figure 2:**
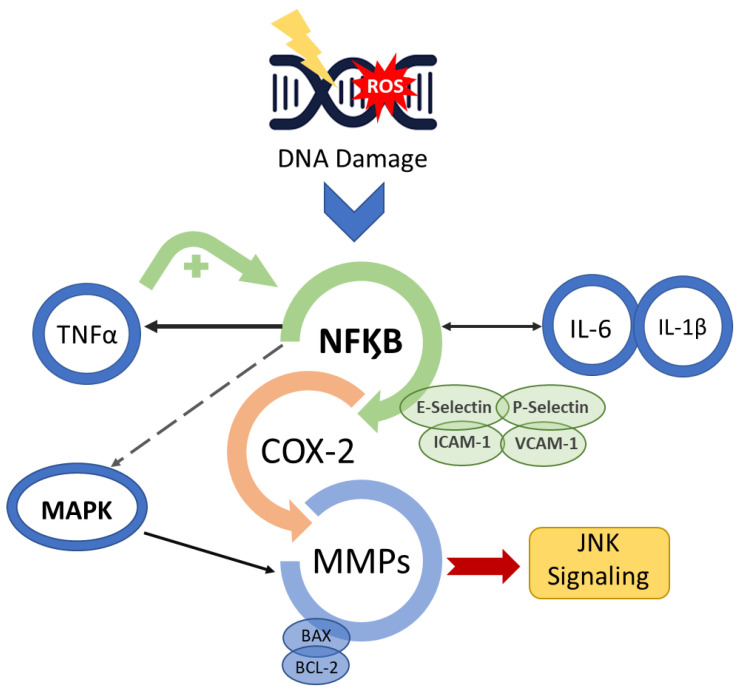
Mucositis signal amplification. Anti-cancer treatment activates the transcription factor nuclear factor-κB (NF-κB) after damage to basal epithelial cells and cells in the underlying tissue. The break in double-stranded DNA, the generation of ROS and PAF release through platelet aggregation leads to cell death and/or injury. NF-κB up-regulation initiates a positive feedback loop and consequently amplifies the production of pro-inflammatory cytokines (TNF-α, interleukin (IL)-6 and IL-1β) as well as the transcription of genes encoding MAPK, COX-2 and tyrosine-kinase signalling molecules, prompting activation of apoptotic genes (BAX and BCL-2) and matrix metalloproteinases.

The genesis of OM in neoplastic treatments occurs with or without mucosal cell DNA damage. Radiation-induced OM takes place when DNA degrades in oral basal-epithelial cells, while chemotherapy-induced OM occurs when basal cells become damaged by chemotherapeutic agents present in the systemic circulation [14]. In radiation-induced OM, reactive oxygen species (ROS) are produced in response to DNA damage. The production of ROS negatively impacts the epithelium by causing irreversible DNA damage, promoting cell apoptosis. It is important to note that, at this stage, patients do not present any clinical symptoms, but the biological disruptions will have already occurred in the submucosa, advancing the progression of OM [13,14,15]. With submucosal injury, initiators of inflammation are triggered (Figure 2). Taken together, these endogenous mechanisms trigger a cascade of biological events and inflammatory pathways that initiate tissue damage in the oral mucosa [5,13,16,17,18].

Additionally, enzymatic and fibroblast activation occurs, further accelerating cell apoptosis in the oral submucosa, advancing OM [19]. The activation of the pro-inflammatory NF-kB pathway also induces the expression of various adhesive molecules, including E-selectin, P-selectin, ICAM-1, vascular adhesion molecule 1 (VCAM-1) and angiogenesis molecules [18,20,21]. Consequently, c-Jun N-terminal kinase (JNK) signalling is triggered, initiating a fibronectin breakdown and leading to macrophage activation [14,15,22,23]. NF-kB also affects genes in the B-cell lymphoma 2 (BCL2) family, which may directly induce cell apoptosis [24].

There is a change in the interface integrity between the epithelium and the submucosa in the basement membrane, which is mediated by the transcription factor that activates protein 1 (AP1), which controls the genes that regulate matrix metalloproteinases (MMP) production [13,14,22,23,24]. The modification in the production of MMP causes destruction of the subepithelial matrix and, consequently, may enhance the effectiveness of other signals, such as those carried out by TNF-α [13].

Tissue-destructive processes result in visible inflammation and ulceration in the oral mucosa, often leading to bacterial colonisation and further aggravating tissue damage. Alterations in the oral microbial communities occur during cancer treatments, leading to microbiota imbalances and consequently to an increase in normally contained populations of microorganisms [25]. The presence of pro-inflammatory microbiota influences the severity of OM. For example, the increased amount of *Porphyromonas gingivalis* and the oral yeasts *Candida glabrata* and *Candida kefyr* alter the recovery capacity of epithelial cells of oral mucosa, leading to a delay in the wound healing capacity [26]. Ruptures in the oral mucosal tissue thereafter led to microbial colonisation and growth, creating a risk of infection for patients. The open wounds in the oral cavity caused by OM allow opportunistic bacteria, such as *Actinomyces, Lactobacillus, Bifidobacterium* and *Eubacterium*, to colonise the tissue and release toxins into the submucosa, causing damage and leading to infection. This also increases the risk of septicaemia in patients with neutropenia [27,28,29].

The inflammatory process culminates with wound healing associated with hematopoietic recovery, the re-establishment of balanced local microbial flora and an absence of factors that interfere with wound healing, such as infection resolution and diminished mechanical irritation [16,30,31]. The resulting repair of the extracellular matrix leads to mucosal renewal and healing via stimulated proliferation, migration, adhesion and differentiation of compromised tissues in the submucosa [15,32]. Nevertheless, residual angiogenesis in the tissue after an OM episode induces higher risk of future episodes [33].

The course of OM progression is not only associated with treatment factors but also with patients’ characteristics such as body mass profiles, renal and hepatic function, local oral factors and genetics [5,31,34]. Despite OM being an independent risk factor for the development of infections, several researchers have demonstrated that microbial flora is not a primary causative factor for the developmental pathology [23]. Once the individual risks for mucositis have been identified, efforts to interfere in the inflammatory responses from different treatment approaches will naturally follow. Therefore, the therapy-based strategy involves the concomitant use of agents that are able to act in different phases of the pathogenesis of mucositis [34].

## 4. Prevention and Management Strategies

OM treatment is a miscellany of therapies that quest the control of the diseases and the symptoms relieved. Therefore, the clinical practice guidelines from the Multinational Association of Supportive Care in Cancer and International Society of Oral Oncology (MASCC/ISOO) summarized the standard protocol to manage the OM in cancer patients [32,34]. The proposed intervention strategy is to begin with the care in oral health with a combination of toothbrushing and flossing [35]. The strategy is followed by the elimination of any type of irritant and the control of the proliferation of oral pathogenic microflora through mechanical removal, combined with the individual use of mouth rinses to maintain oral hygiene. Complete oral examinations and dental interventions are critical components performed in conjunction with oncological treatments [32,34,35].

Oral cryotherapy and photobiomodulation (PBM) therapy have been utilised preventively to reduce the impact of the treatment toxicity in the oral mucosa [36]. The PBM is recommended for the prevention and treatment of OM in patients receiving cancer treatments. Several studies have demonstrated the effectiveness of anti-inflammatory effects in supporting tissue repair [13,33,37,38,39,40,41,42,43,44,45]. Nevertheless, clinical evidence still shows that some patients present recurring episodes of OM during their cancer therapy despite being treated with LLLT [37].

Pharmacological agents (pentoxifylline, benzydamine hydrochloride, thalidomide and simvastatin) currently utilized to prevent and treat OM have variable efficacy rates and significant side effects, rendering this treatment strategy less than ideal [13,14,15]. The requirement to reduce the side effects of pharmacological agents and increase the possibility of a patient’s fast recovery elicited the need for research to demonstrate the benefits of utilising natural resources and herbal medicines to manage the OM wound and related inflammatory conditions [3,33]. For this reason, several natural products such as chamomile, essentials oils from manuka (*Leptospermum scoparium*) and kanuka (*Kunzea ericoides*), vitamins A, B12 and E, folate, glutamine, aloe vera and curcumin have been studied [46,47,48,49]. In addition, several studies have investigated the mechanisms of action of n-3 fatty acids (or omega-3 fatty acids) against several diseases, with observed successes that are likely due to the fatty acids’ anti-inflammatory effects [50].

Researchers have demonstrated that the use of a combination of agents and physical strategies can provide anti-inflammatory, analgesic and anti-microbial effects that can be used to manage cancer-therapy-induced OM in general. The combination strategy has been promising for patients’ symptom relief and wellness during the OM course [13,37].

## 5. Omega-3: Inflammation Reduction and Tissue Homeostasis Recovery

**Figure 3:**
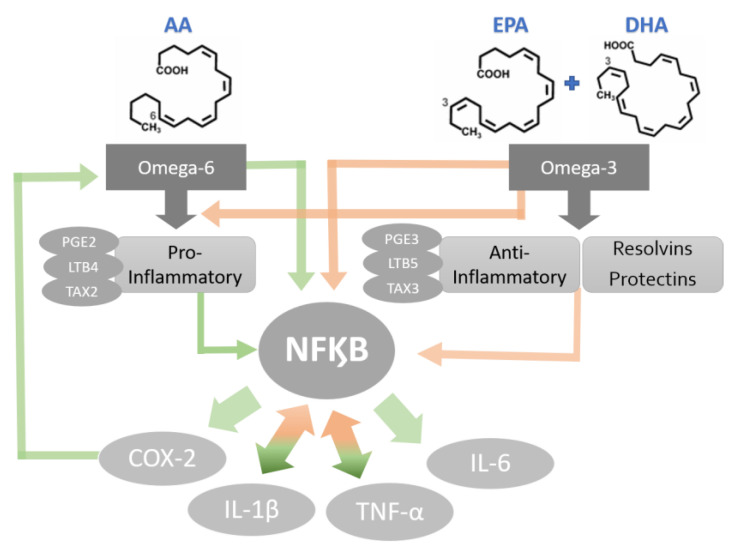
Potential mechanisms of PUFA-activated cytokine production. Green bold arrows indicate activation or up-regulation and red bold arrows represent inhibition or down regulation.

The PUFAs are a part of the group of fats (lipids) that are the main components of cellular membranes [51]. The shift in cell membrane compositions could be the mechanism of anti-inflammatory agents as well as immune cell activations [52,53]. The synthesis of eicosanoids (prostaglandins, prostacyclins, thromboxanes and leukotrienes) is generated by the presence of omega-3 (eicosapentaenoic acid (EPA; C20: 5ω-3), docosahexaenoic acid (DHA; C22: 6ω-3)) and omega-6 (arachidonic acid (AA)) incorporated into the cell membrane [53]. Further, metabolic enzymes act on EPA and DHA, producing metabolites that activate other anti-inflammatory pathways and weaken the inflammatory action of immune cells [54,55] (Figure 3).

Omega-3 PUFAs have been proven to inhibit inflammatory processes in several ways. The blockage of the AA cascade is an anti-inflammatory mechanism frequently studied as the pathway is linked with the daily omega-3 (EPA and DHA) supplementation [56,57,58,59]. The increased bioavailability of omega-3 leads to an additional level of immunoregulation by these FFAs [56,57]. Publications show that AA metabolites, such as prostaglandin and leukotrienes, are involved in oral health and diseases, including the modulation of salivary gland inflammation [60]. Although the role of AA metabolites in OM is not well established, there is evidence that topical prostaglandin-E2 (PGE2) application is effective in reducing chemoradiotherapy-induced OM [61]. Therefore, EPA and DHA used to reduce the oral mucosa inflammatory process should be further investigated to elucidate whether the FFAs promote inhibition of pro-inflammatory cascades during the OM development. The action of PUFAs on the immune system is related not only to the profile alteration of eicosanoids during inflammatory activation but also to the alteration of pro-inflammatory protein production, including cytokines and adhesion molecules [56].

A prominent anti-inflammatory mechanism of action of omega-3 is its epigenetic inhibition of NF-kB in response to inflammatory stimuli [59]. This epigenetic alteration is also responsible for the decrease in the expression levels of adhesion molecules, inflammatory cytokines and COX-2 metabolites [62,63]. In addition, FFA can block the translocation of NF-kB to the nucleus by inducing peroxisome proliferator-activated receptor gamma (PPARg) target genes [64]. This translocation inhibition leads to a decrease in the production of cytokines such as TNF-α, IL-1β and IL-6 [55,62,63,64]. According to Calder (2013), the omega-3 FFAs elicit an effect on inflammatory genes’ expression via the inhibition of the activation of the transcription factor NF-kB in response to exogenous inflammatory stimuli [63].

As previously mentioned, NF-kB is a primary driver in the process of mucositis pathobiology and the severity of the disease is associated with the pathway activators. Their activation leads to further production of the principal inflammatory target cytokines, which, in turn, amplifies the response [18]. Omega-3 blocking the NF-kB migration to the nucleus could decrease the production of cytokines responsible for the positive feedback and affect the development of the OM signal amplification phase. The blockade of this stage alters the biological environment to reduce the message generation that activates the damaged response pathways. Consequently, there would be a stop in the cytokines’ feedback loops and a slowdown of tissue injury. However, as the source of damage remains due the chemotherapeutic or irradiation insult, the mucosa still presents molecular alterations that would delay the total tissue recovery until the end of oncological treatment.

In addition to acting in several pathways, omega-3 FFA modulates immune cells’ activity. PUFAs act directly on several immune cells. FFA supplementation modulates neutrophil function, including migration and phagocytic capacity as well as the production of reactive oxygen species and cytokines. It stimulates macrophage cells to produce and secrete cytokines and chemokines, to increase capacity of phagocytosis and the cells polarization and to modulate T cell activation [55].

Mucosa wound recovery in cancer patients could be supported by the daily intake of these natural supplements, not only to increase the immune cell’s capacity to act directly in the mucosa but also in the systemic immunoregulation. During the OM development, there is an activation of inflammatory cascades that affect mast cells, neutrophils and natural killer cells, leading to the production of AA metabolites, toxic phagocytic products (oxygen metabolites, nitric oxide, collagenases, etc.), toxic lymphocyte products, neuropeptides and various components of the plasma proteolytic cascades [65]. In this condition, FFAs would be able to reduce inflammation through immune cell regulation and support wound reepithelialisation.

## 6. Evidence of Omega-3 as Therapeutic Strategy

Natural and herbal remedies have been precursors to numerous medicines that are commercialised and utilised today [66]. A novel strategy to speed up the recovery of OM is to improve patients’ nutrition by increasing their intake of omega-3. This nutritive change may be particularly effective in promoting tissue recovery, decreasing inflammation and improving the body’s natural immune response to OM [67]. The anti-inflammatory and healing properties of omega-3 fatty acids make them exciting potential pharmaceutical agents for several pathological conditions. Evidence suggests that local and systemic levels of inflammatory mediators, combined with EPA and DHA oral supplementation, may encourage inflammation resolution, PMN down-regulation and wound reepithelialisation [68].

In a critical review, the anti-inflammatory potential of the compounds has also been evaluated in the management of different cancer therapy side effects, such as anorexia-cachexia syndrome, pain, depression and paraneoplastic syndromes. The authors concluded, through preclinical evidence, that omega-3 PUFAs and their metabolites might modulate the main pathways underlying complications secondary to cancer [69]. The evidence that PUFAs are promising for handling the toxicity effects of the oncologic treatment is an initial point to evaluate their use in the OM prevention and treatment.

Although omega-3 may be a promising agent to treat and manage various pathologies, there are few studies that investigate how and if omega-3 fatty acids aid in the healing of wounds from different aetiologies (Table 1). Omega-3 activity in the recovery of epithelial cells has been demonstrated in clinical trial studies. The FFA capacity to reduce swelling and pain without debridement of the necrotic tissue in cutaneous wound healing was demonstrated. In addition, the studies have shown that FFA increases proinflammatory cytokine production, PMN down-regulation and wound reepithelialization [56,68]. Although the process of wound healing in the oral mucosa is starkly different to other tissues, the evidence from the studies gives a broad sense for the way that omega-3 could act in mucosal recovery.

Animal studies have demonstrated that supplementing the diet with 0.2% to 0.4% of the total animal weight with omega-3 was associated with accelerating ulcer healing in murine mucosal models [70,71]. Hashemipour et al. concluded that omega-3 was effective in wound re-epithelialization, increasing the average thickness of the epithelium and encouraging inflammation resolution [71]. The use of FFA in mucosal recovery also specifically increases the formation of granulation tissue, encourages fibroblast action and reduces the severity and size of oral wounds [70,71]. Despite both studies having been conducted using animal models, the results agree with the general understanding of the mechanism action of omega-3 in tissue inflammatory processes.

Two double-blind, placebo-controlled mucosa cell-based clinical trials assessed the effects of the systemic use of omega-3 on the treatment of recurrent aphthous stomatitis. Both studies note that omega-3 treatment achieved a significant reduction in ulcer numbers, duration in the tissue and level of pain. Results indicate that the daily consumption of omega-3 capsules of 1000 mg (200 mg of DHA and 300 mg of EPA essential fatty acids) could be effective in the management of recurrent ulcers in the oral cavity [72,73]. Even though there are differences in etiopathology between aphthous stomatitis and OM, the study’s conclusion brings forth important considerations regarding mucosa recovery under omega-3 influence.

In one clinical study, patients received 2000 mg of fish oil (360 mg of EPA and 240 mg of DHA) omega-3 fatty acids and presented significantly less pain and irritation of the oral cavity [74]. Despite the limitations of this particular study, the clinical observations support the premise that dietary intake of omega-3 can affect molecular and cellular activities supporting tissue recovery [59]. Several separate experiments have also demonstrated the positive effects of omega-3 fatty acids in preventing complications associated with diseases and treatments across a range of cyclosporine use, hypertension, diabetes, arthritis, other inflammatory conditions, autoimmune disorders and cancer [54,59].

The American Heart Association (AHA) has suggested that a safe EPA and DHA dosage for healthy people is 0.5 g to 1.8 g daily [75]. This is equivalent to approximately one to two servings of fish per week. However, this recommendation is based on the calculated effective dosage for preventing cardiovascular diseases and their associated mortality. Specific dosage recommendations for the treatment of OM must be established before this treatment strategy is clinically implemented. Furthermore, AHA guidelines advise monitoring patients who consume high doses of EPA and DHA (>3 g/d) because of the potential complication of excessive bleeding [75]. Additionally, the AHA cautions consumers to be aware of their fish sources, as some species of fish contain high concentrations of toxins, such as methylmercury and dioxins [75]. This risk can be mitigated by consuming younger and smaller fish and consuming fish from low-risk waters. Generally, the benefits of consuming omega-3 fatty acids appear to outweigh the risks. Therefore, omega-3, in safe doses, could be a feasible intervention to aid in the recovery of OM. The increased use of natural medicine instead of the synthetic pharmaceuticals in the management of some diseases is mainly due to less adverse effects [76]. Studies have demonstrated the possibility of handling the healing of different wounds via the dietary intake of PUFAs based on their anti-inflammatory effects [56,68,70,71,72,73,74]. Altogether, it is possible to manage OM via the dietary intake of PUFAs due to their anti-inflammatory effects. In short, the anti-inflammatory mechanisms of PUFAs include decreasing immune cell recruitment, switching pro-inflammatory pathways and eliminating apoptotic cells through phagocytosis, resulting in tissue healing [76].

This review outcome agrees with the latest research findings, that PUFAs present a promising approach in addressing the lack or delay of the recovery of OM during cancer treatment, possibly improving the overall quality of life and, consequently, patients’ survival [77].

## 7. Conclusions

Evidence suggests that the dietary supplementation of FFA could be effective for managing the complications associated with cancer treatment, including the physiological development of OM. This intervention is especially relevant for cancer patients not responding to current OM treatment strategies. Nevertheless, this possibility needs to be better studied before it can be clinically implemented. Studies on the pathophysiology of OM, including its pharmacogenomic influences and epidemiology, will be helpful to better understand the disease. Meta-analyses on the best pharmaceutical agent combinations to treat OM will also be beneficial in this pursuit. Importantly, more primary research must be conducted on the anti-inflammatory mechanisms of omega-3 fatty acids to discern if this supplementation strategy will truly be efficacious for treating inflammatory diseases such as OM; if so, then the optimised dosage regime of omega-3 fatty acids for OM must be discovered before clinical implementation is possible.

## Figures and Tables

**Table 1:** Summary of the characteristics of studies investigating the effectiveness of omega-3 (ω-3) PUFAS on different ulcers.

References	Study Description		Omega-3 Dose	Key Finding
McDaniel et al. 2008 [56]	Clinical Trial, Randomized and Double-blind Control Study.	Evaluation of plasma fatty acid levels in healthy individuals (*n* = 30) at baseline and after 4 weeks in a blister wound model.	ω-3 group: total daily intake of 1.6 g of EPA and 1.1 g DHA capsules daily/4 weeks.	PUFA may increase proinflammatory cytokine production at wound sites at 24 h and non significantly slower wound healing.
McDaniel et al. 2011 [68]	Clinical Trial, Randomized and Double-blind Control Study.	Evaluation of lipid mediator levels in acute wound and the reduction of PMN levels in healthy individuals (*n* = 18) by a blister wound model.	Active group: 1.6 g of EPA and 1.2 g of DHA per day/28 days.	ω-3 group presented lower mean levels of myeloperoxidase at 12 h and more reepithelialisation on Day 5 post wounding.
Abdelsalam et al. 2017 [70]	Murine model, Group-control Study	2 groups (*n* = 15 per group). Histological samples harvested on post-injury days 3, 7 and 14.	Systemic: 93 mg/kg body weight	The study group had high reepithelialisation and connective tissue healing score on day 7 and 14.
Hashemipour et al. 2012 [71]	Murine model, Group-control Study	5 groups (*n* = 16 per group). On post-injury days 2, 4, 6, and 8, tissues harvested for histological evaluation.	Local: 100 mg/kg (0.2% total weight) and Systemic: 200 mg/kg (0.4% total weight)	The control group had highest inflammation, and the lowest reepithelialisation. The thickest epithelium was observed in the local and systemic groups on days 6 and 8.
El Khouli & El-Gendy 2014 [72]	Clinical Trial, Randomized and Double-blind Control Study.	Patients diagnosis with recurrent aphthous ulcer (*n* = 50). Evaluation by number of new ulcers, duration of ulcer episodes, and pain level through questionnaires.	Experimental group: ω-3 (1 g-200 mg of DHA and 300 mg of EPA), 3x daily/6 months.	Daily ω-3 treatment achieved a significant reduction in number of ulcers, duration of ulcers, and level of pain by 3 months that persist for 6 months.
Nosratzehi & Akar 2016 [73]	Clinical Trial, Randomized and Double-blind Control Study.	Patients diagnosis with recurrent aphthous ulcer (*n* = 50). Size and rate of ulcers was measured weekly.	ω-3 group: 1 g of DHA and EPA, 3 times daily for 6 months.	The ω-3 group present less pain and irritation. The ulcer size decrease from 2.3 to 1.48 mm (*p* = 0.062). The number of ulcers indicates a reduction in comparison with the control group.
Hashemipour et al. 2017 [74]	Clinical Trial, Randomized and Double-blind Control Study.	Patients with leukaemia or breast cancer diagnosis undergoing chemotherapy treatment that developed oral mucositis (*n* = 60). Oral examinations were repeated on days 1, 7, 14, and 21.	ω-3 group: 1 g pearl (360 mg of EPA, and 240 mg of DHA), 2 capsules daily.	Differences in the severity of mucositis and pain score between the ω-3 and placebo groups in the first, second, and third weeks of treatment were noted.

## Abbreviations

AA	Arachidonic acid
AHA	American Heart Association
ALA	Alpha-linolenic acid
AP1	Activator protein 1.
BAX	BCL-2-like protein 4
BCL2	B-cell lymphoma 2 gene
COX-2	Cyclooxygenase-2
DHA	Docosahexaenoic acid
DNA	Deoxyribonucleic acid
EPA	Eicosapentaenoic acid
FFA	Free fatty acids
GPx	Glutathione peroxidase
HO-1	Heme oxygenase 1
HSCT	Hematopoietic stem cell transplantation
ICAM-1	Endothelial- and leukocyte-associated transmembrane
ICU	Intensive care unit
IL-1β	Interleukin 1 beta
IL-6	Interleukin 6
JNK	c- Jun N-terminal kinase
LLLT	Low-level laser therapy
MAPK	Mitogenated protein kinase
MASCC/ISOO	The Mucositis Study Group of the Multinational Association for Supportive Care in Cancer and the International Society of Oral Oncology
MMP	Matrix metalloproteinase
MTX	Methotrexate
NF-kB	Nuclear factor-kape Beta
OM	Oral mucositis
ω-3	Omega-3PAF: Platelet-activating factor
PBM	Photobiomodulation
PMN	Polymorphonuclear leukocytes
PPARg	Peroxisome Proliferator Activated Receptor Gamma
PUFA	Polyunsaturated fatty acids
ROS	Reactive oxygen species
TNF-α	Tumour necrosis factor alpha
TPN	Total parenteral nutrition
U.S. FDA	United States Food and Drug Administration
VCAM-1	Vascular adhesion molecule 1
